# Facile synthesis of ZnO/Hal nanocomposite for arsenite (As(III)) removal from aqueous media

**DOI:** 10.1038/s41598-023-48531-5

**Published:** 2023-12-02

**Authors:** Mohammad Ali Khoddam, Reza Norouzbeigi, Elmira Velayi, Giuseppe Cavallaro

**Affiliations:** 1 Nanomaterials and Surface Technology Research Laboratory, School of Chemical, Petroleum and Gas Engineering, Iran University of Science and Technology, Narmak, P.B. 16765–163, Tehran, Iran; 2 Department of Chemical Engineering, Faculty of Engineering, Azarbaijan Shahid Madani University, P.O.Box: 537517–1379, Tabriz, Iran; 3 Dipartimento di Fisica e Chimica, Università degli Studi di Palermo, Viale delle Scienze, pad. 17, 90128 Palermo, Italy

**Keywords:** Environmental sciences, Nanoscience and technology

## Abstract

Arsenite (As(III)) is the most toxic form of arsenic that is a serious concern for water contamination worldwide. Herein a ZnO/Halloysite (Hal) nanocomposite was prepared by the chemical bath deposition method (CBD) through seed-mediated ZnO growth on the halloysite for eliminating As(III) from the aqueous solution. The growth of ZnO on seeded halloysite was investigated based on the HMTA: Zn^2+^ molar ratio in the solution. An optimum molar ratio of HMTA:Zn for nucleation and growth of ZnO upon halloysite was obtained 1:2 based on morphological analysis. The TGA results confirmed that thermal stability of HNT was enhanced by ZnO decoration. The prepared ZnO/Hal nanocomposite at optimal conditions was employed for arsenite (As(III)) removal from aqueous solutions. Experimental data were evaluated with different isothermal, thermodynamic, and kinetic models. Based on the zeta potential results, Hal nanocomposites had a greater negative value than pure Hal. Therefore, the ZnO/Hal nanocomposite exhibited efficient As(III) adsorption with a removal efficiency of 76% compared to pure Hal with a removal efficiency of 5%. Adsorption isotherm was well correlated by both non-linear Langmuir and Sips models, exhibiting maximum adsorption capacity of As(III) at 42.07 mg/g, and 42.5 mg/g, respectively. As a result of the study, it was found that the fabricated Hal nanocomposite with low toxicity can be used effectively in water treatment.

## Introduction

Controlling heavy metal pollution in drinking and underground water is one of the most significant challenges in the world. Among the heavy metals, arsenic is a highly toxic metal that can cause various problems for human health^1,2^. The maximum concentration of arsenic in water sources is 10 ppb, according to the World Health Organization (WHO). In general, arsenic is classified chemically as a metalloid that is too mobile in the environment, which is directly related to arsenic's oxidation state and parent mineral content. In the classification of arsenic based on the oxidation state, it is divided into arsenite (As(III)), arsenate (As(V)), arsenic (As(0)), and arsine (As(III)). The dominant types are arsenite (As(III)), and arsenate (As(V))^3,4^. Arsenite (As(III)) is a more toxic and mobile species than arsenate (As(V)). It isn’t easy to eliminate it because of uncharged species in natural water. Based on the literature, arsenite is 60 times more toxic than arsenate^5,6^. It should be noted that the determination of the removal method depends on the toxicity of the mentioned pollutants. Up to now, numerous methods have been reported for removing arsenite from an aqueous solution including oxidation, membrane technology, co-precipitation and coagulation, and ion exchange^3,7–9^. These methods may have a high ability in arsenic removal but also have some disadvantages like high operational costs, low efficiency, production of toxic by-products (co-precipitation and coagulation)^10^, and usually requiring secondary treatment^11–13^. Moreover, some crucial factors such as simplicity, easy operation, and maintenance, availability and flexibility, low price, and non-toxicity should be considered in selecting the practical method for pollutant removal. There are many advantages to adsorption as a method of removing various pollutants from wastewater, including cationic and anionic organic dyes^14^, heavy metal ions such as Cr(VI)^15^ and As(III)^16,17^, including the fact that it is inexpensive, does not involve undesirable products, and doesn't require complicated equipment. Moreover, the adsorption method is commonly considered environmentally friendly because the sorbents can be regenerated and reused several times. Tremendous adsorbents have been used to remove arsenic such as polymers^18^, biomaterials^17^, clays^19^, metal organic framework (MOF)^20^, magnetic composites^21^, and ion exchange resins and minerals. Due to the intra-particle diffusion phenomenon in macromolecules, which causes a decrease in adsorption rate and capacity, researchers have considered nanostructure adsorbents^22,23^. For example, Salama et al.^24^ investigated the use of nano zero-valent iron supported on silica gel for simultaneous Cr(VI) removal. In another study, cationic and anionic dyes were removed from aqueous solutions using citric acid functionalized nickel–cobalt sulphide nanoparticles^25^. Nanoclays are natural materials with attractive properties, including broadly accessible, low-price, high specific surface area, suitable porosity, layered structure, non-toxic, and proper functionalization. These materials are suitable adsorbents for arsenic removal; some of them can be directly used for arsenic removal, and the rest need to be activated^26^. Halloysite nanotube (Hal) is a natural 1:1 aluminosilicate mineral with a length of 7–10 µm, an inner diameter of 10–15 nm, and an external diameter of 40–60 nm, which is chemically similar to kaolinite. It has a unique and attractive tubular structure with active sites on the inner surface (Al–OH) and outer surface (Si–O–Si) with different electric charges^27,28^. Because of these specific properties like mesoporous lumen and the surface functional groups, halloysite can be used as an applicable host for supporting nanoparticles in heavy metal removal^29^. To our knowledge, limited research has been conducted on arsenic removal using halloysite. Different types of halloysite nanocomposites such as halloysite–CeOx nanohybrid^30^, Hals/C/Fe_3_O_4_^31^, Fe_3_O_4_@SiO_2_@Mn-Hal^32^, Fe_3_O_4_@Hal^33^ are used for arsenic removal. Song et al.^30^ synthesized halloysite-CeO_x_ nanocomposites using a redox-precipitation method to remove As(III). The formation of surface complexes and oxidation of As (III) to As(V) followed by adsorption of As(V) was described as the adsorption mechanism of As(III). To remove arsenic from water, Song and coworkers^34^ created cactus-like Fe_3_O_4_/Hallosyte nanocomposite using a coprecipitation method. The cactus-like Fe_3_O_4_ exhibited excellent reusability with an arsenic removal efficiency higher than 80% after 6 reuse cycles. Although many studies have been conducted on utilizing halloysite nanocomposites for heavy metal removal, using ZnO-halloysite nanocomposites to remove arsenic from water has attracted little attention from researchers. The novel aspect of this study is the decoration of nontoxic ZnO onto halloysite and the optimization of the adsorption conditions for the removal of As(III) from aqueous solutions via ZnO/Hal.

This investigation aims to fabricate a non-toxic nanocomposite with antibacterial and functional properties for arsenic removal. It is common to decorate nanoparticles on the support to enhance their performance and ability to adsorb contaminants. Due to its easy dispersion in water and reusability, halloysite is a more cost-effective and cheaper alternative to carbon nanotubes. For this reason, a zinc oxide/halloysite nanocomposite was fabricated by the chemical bath deposition (CBD) method in this research. Fourier-transform infrared spectroscopy (FTIR), X-ray diffraction (XRD), field emission scanning electron microscopy (FESEM), transmission electron microscopy (TEM), scanning transmission electron microscopy (STEM), surface area analysis (BET), thermal gravimetric analysis (TGA), and zeta potential analyses were used to characterize the morphology and chemical composition of the prepared nanocomposite, and then the arsenic removal efficiency was studied.

## Material and method

The Hal was provided by Sigma-Aldrich. The Hals had a diameter of 30–70 nm, and their lengths were in the range of 1–3 µm. Zinc acetate dihydrate [Zn(O_2_CCH_3_)_2_·2H_2_O] was purchased from Across Company. Hexamethylenetetramine (HMTA, C_6_H_12_N_4_), and ethanol (C_2_H_6_O) were purchased from Merck Company.

### Preparation ZnO@Hal

ZnO@Hal was prepared by the dip coating approach via a solution of zinc acetate. Firstly, zinc acetate dihydrate was dissolved in deionized water (0.11 M, Solution A), and sonicated for 15 min. After that, Hal was added (12.5 g/L) to solution A under constant stirring for 24 h, and the pH level was maintained at 6–7. Then precipitates were washed with deionized water and ethanol several times and dried in a vacuum oven at 60 °C for 4 h. The dried precipitates were then calcinated for 2 h at 500 °C. The resulting products were Hals@ZnO^35^.

### Growth of ZnO upon ZnO@Hal

The CBD method was applied to the growth of ZnO upon ZnO@Hal. HMTA solution was used as a complexing agent. Firstly, a zinc nitrate hexahydrate (Zn (NO_3_)_2_.6H_2_O) solution (0.025 M) was prepared by dissolving the specified amount of zinc nitrate in deionized water (Solution B). Afterward, HMTA was mixed with solution B at different molar ratios of 1:1 and 1:2 under constant stirring. Subsequently, the solutions were transferred to a sealed glass bottle and heated in an oil bath at 90 ± 5 °C for 2 h^36,37^. The solution was filtered and washed at least three times with deionized water. The collected powder was dried at 150 °C for 1 h. The obtained material is identified as ZnO/Hal samples in the following paragraphs.

### Characterization

The crystallographic investigation of prepared samples was conducted by an X-Ray diffractometer (Netherland, PHILIPS, PW1730) using nickel-filtered Cu Ka radiation (k = 1.5418 Å). The chemical composition of samples was estimated using FTIR (USA, THERMO, AVATAR model). The aqueous arsenic concentration was recorded via ICP-AES (USA, VARIAN, 730-ES). Morphological features of the samples were evaluated by field emission scanning electron microscopy coupled with an energy dispersive spectrometer (FE-SEM, TESCAN, MIRA III), scanning transmission electron microscopy (STEM, THERMOSCIENTIFIC, QUATTRO S SEM) and transmission electron microscopy (TEM, Philips EM208S 100 kV, Netherland). A thermogravimetric analyzer (Instrument TGA Q5000 V3.17) was used to characterize the thermal decomposition of pure Hal, ZnO@Hal, and ZnO/Hal. The Zeta potential values of the nanocomposites were measured using a zeta potential instrument (Japan, Horiba, SZ100). Each data point presents the mean value of three measurements at 25 °C.

### Arsenic adsorption test

Bach adsorption experiments were performed at ambient temperature using 100 mL sealed glass bottles. The 50 ± 0.2 ppm concentration of As(III) solution was prepared by dissolving a proper amount of NaAsO_2_ in deionized water. The adsorbent dosage of 2.5 g/L, shaking speed of 120 rpm, and pH of 6.5 ± 0.5 were considered for the adsorption experiments. After 24 h, the adsorbents were separated via a centrifugation process, and the residual concentration of As(III) was measured using an inductively coupled plasma optical emission spectrometer (ICP-OES, VARIAN VISTA PRO). The equilibrium adsorption capacity and removal efficiency were determined by the following equations

1 $${q}_{e}=\frac{\left({C}_{0}-{C}_{e}\right)\times V}{m}$$

2 $$Removal\%=\frac{\left({C}_{0}-{C}_{e}\right)}{{C}_{0}}$$

C_o_ and C_e_ are the initial and equilibrium concentrations of As(III) (mg/L), respectively. V is the solution volume (L), m is the adsorbent mass (g) and q_e_ is the equilibrium adsorption capacity (mg/g).

## Result and discussion

### Effect of molar ratio of HMTA: Zn^2+^

**Figure 1:**
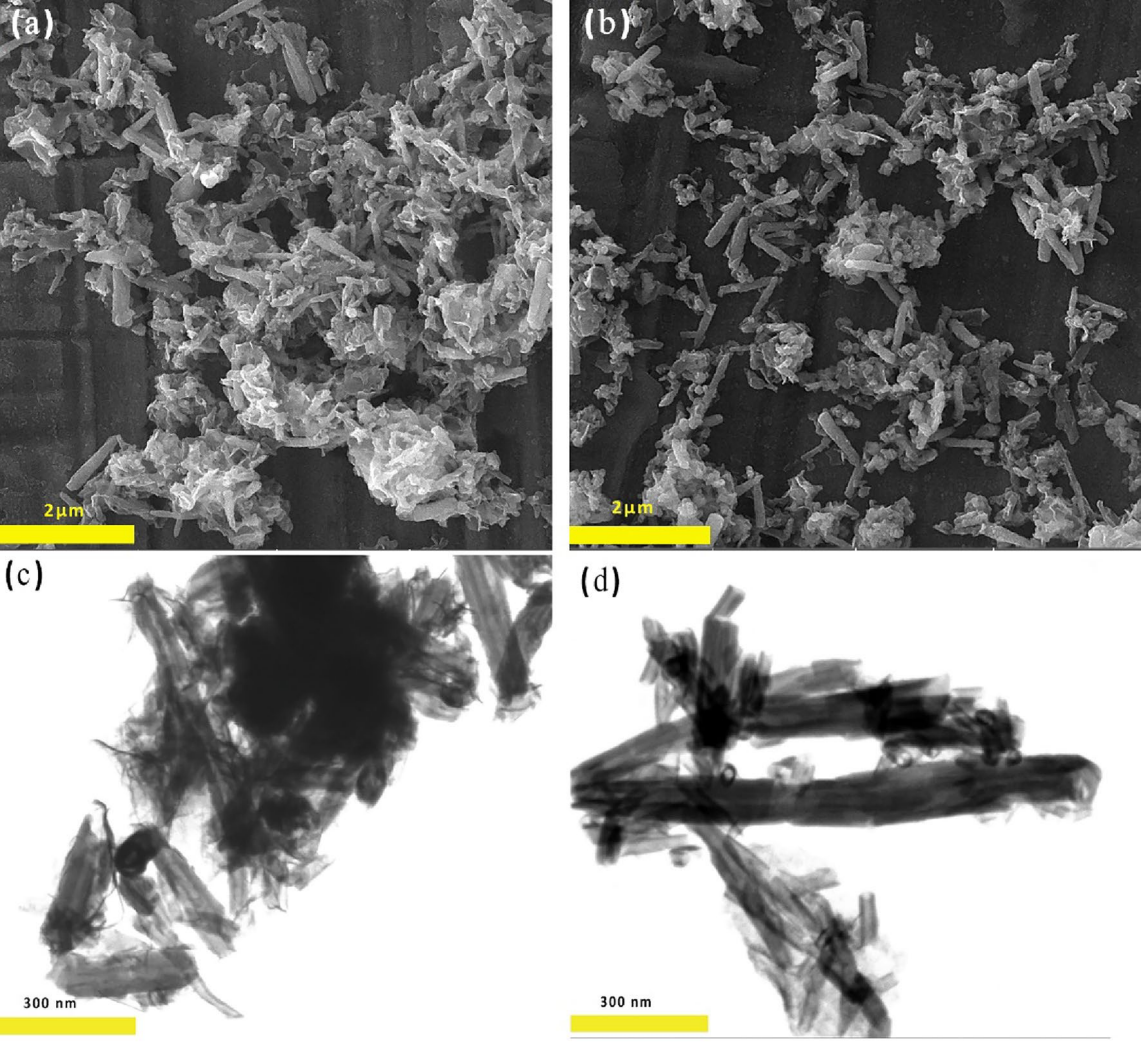
STEM and FESEM photos of ZnO/Hal nanocomposite in the different molar rates of HMTA: Zn^2+^, (**a**, **c**) 1:1 and (**b**, **d**) 1:2.

Two processes occur during ZnO deposition: a heterogeneous reaction on the halloysite results in a ZnO film, and colloidal ZnO particles form in the bulk solution. A critical parameter that controls the desired heterogeneous nucleation of ZnO and its growth on halloysite is the molar ratio of HMTA to Zn^2+^ in solution. The FESEM and STEM results in Fig. 1a–d confirmed an increase in homogeneous growth and colloidal particle formation by increasing the HMTA to Zn^2+^ molar ratio. It is suggested that the desired nucleation and growth of ZnO upon halloysite occurred using a molar ratio of 1:2 as the optimum molar ratio. XRD, FESEM, TEM, FTIR, zeta potential, and BET analyses were used to investigate physiochemical properties variations of Hal after coating with ZnO (coated at the optimal molar ratio of HMTA to Zn^2+^ (1:2)).

### XRD analysis

**Figure 2:**
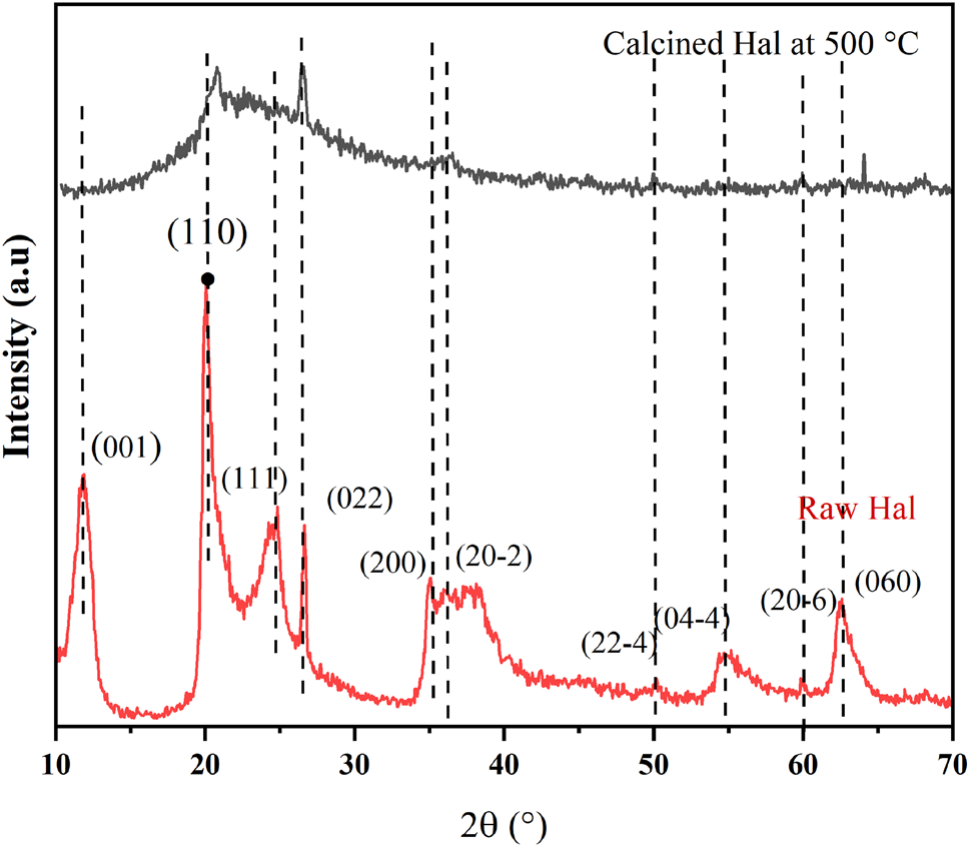
The XRD patterns of raw Hal and calcined Hal at 500 °C.

**Figure 3:**
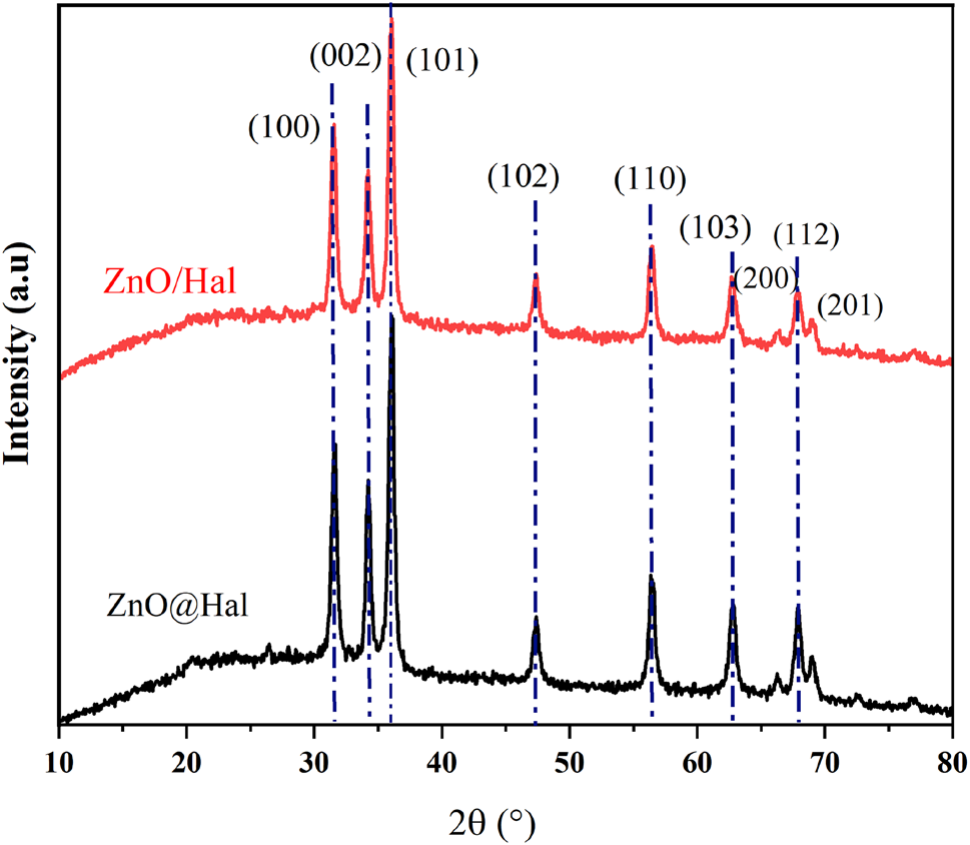
XRD patterns of ZnO@Hal and ZnO/Hal nanocomposite.

The crystalline structure of halloysite nanotubes, ZnO@Hal and ZnO/Hal nanocomposite has been investigated by XRD analysis. As shown in Fig. 2, the observed peaks at 2θ of 19.97°, 23.04°, 24.6°, 26.65°, and 35.06° are attributed to halloysite's crystalline structure according to JCPDS card no. 09-045. After the calcination process at 500 °C, obvious decreases in some peak intensity were observed and some of them disappeared, which might be to the dihydroxylation and dehydration of the halloysite structure (Fig. 2)^38^. Generally, calcination at higher temperatures destroys the halloysite structure due to dihydroxylation and the formation of an amorphous meta-halloysite phase. The XRD patterns of ZnO@Hal and ZnO/Hal are shown in Fig. 3. As can be seen in Fig. 3, the obvious diffraction peaks appeared at 2θ of 31.7°, 34.4°, 36.2°, 47.5°, 56.5°, 62.8°, and 67.8°. These signals can be ascribed to the wurtzite hexagonal structure of ZnO (JCPDS card no. 36-1451), which indicates that the prepared ZnO had high crystallinity and purity. The XRD patterns of the ZnO@Hal and the ZnO/Hal exhibited peaks similar to ZnO, which may be due to the high crystallinity and high mass ratio of ZnO to Hals. Additionally, a comparison of the XRD spectra indicated that the diffraction peaks of ZnO in the spectrum of ZnO@Hal were slightly sharper than that of ZnO/Hal, which reveals a decrease in the crystallinity of ZnO.

### FTIR studies

**Figure 4:**
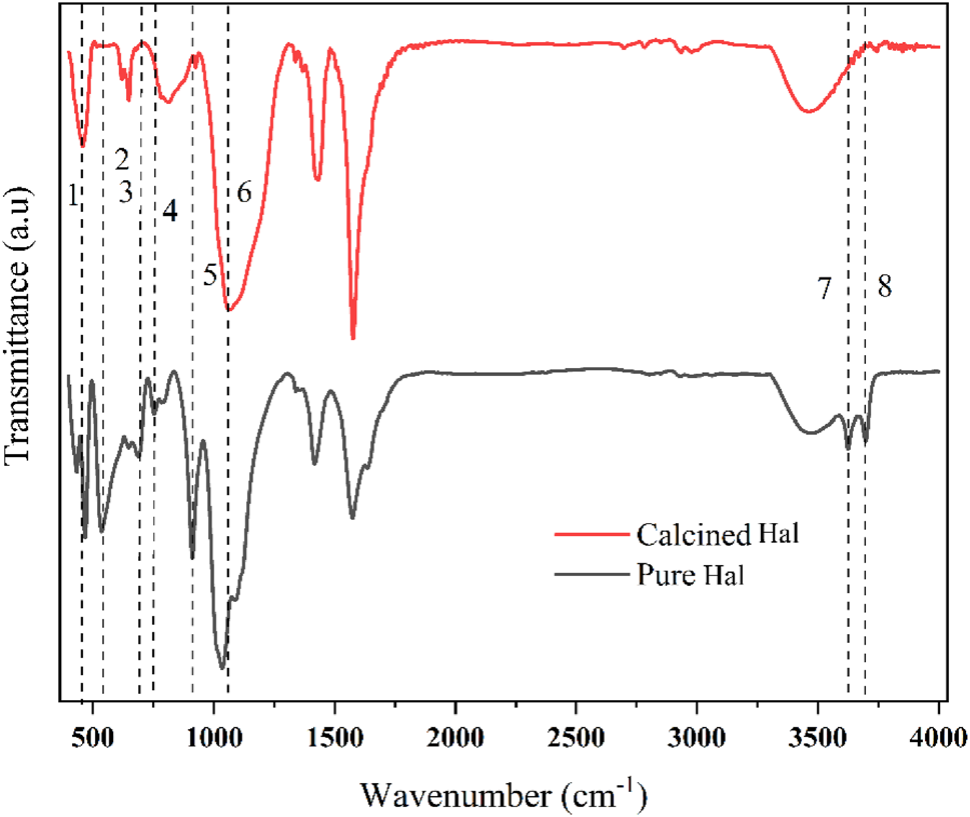
FTIR spectra of the Hal and the calcined Hal at 500 °C.

FTIR studies were used to characterize the chemical structure of Hal and the study the successful modifications made to it. The FTIR results of Hal before and after calcination at 500 °C are given in Fig. 4. The absorption bands related to the functional groups are summarized in Table 1. As observed in FTIR spectra the absorption band at 470 cm^−1^, which is assigned to the deformation of Si–O–Si, is broadened and shifted gradually to the lower wavenumbers with increasing the calcination temperature up to 500 °C. The intensity of the Al–O–Si deformation band (546 cm^−1^) was significantly reduced for the calcined sample at 500 °C, indicating that dihydroxylation was accompanied by the destruction of the Al–O–Si linkages of the halloysite. Additionally, the Si–O perpendicular stretching vibrations, which can be seen at 690, 756, and 1102 cm^−1^, are decreased in the FTIR spectrum of the calcined sample because of the destruction of the ordered Si_2_O_5_ network. It can be concluded that calcination of Hal at temperatures more than 500 °C results in the formation of meta-halloysite. The variations in the peak position of FTIR are clearly illustrated in Table 1 and Fig. 4^38–40^.

**Table 1:** Positions and assignments of the IR vibration bands.

Peak no.	Wavenumber (cm^−1^)		Band assignments
	Hql	Calcined Hal at 500 °C	
1	470	465	Deformation of Si–O–Si
2	546	542	Deformation of Al–O–Si
3	690	694	Perpendicular Si–O stretching
4	756	757	Perpendicular Si–O stretching
5	912	904	Al–OH deformation of inner hydroxyl groups
6	1034	1040	1028 to 1096—in-plane Si–O–Si stretching
7	3626	3619	O–H stretching of inner hydroxyl groups
8	3700	3693	O–H stretching of inner-surface hydroxyl groups

**Figure 5:**
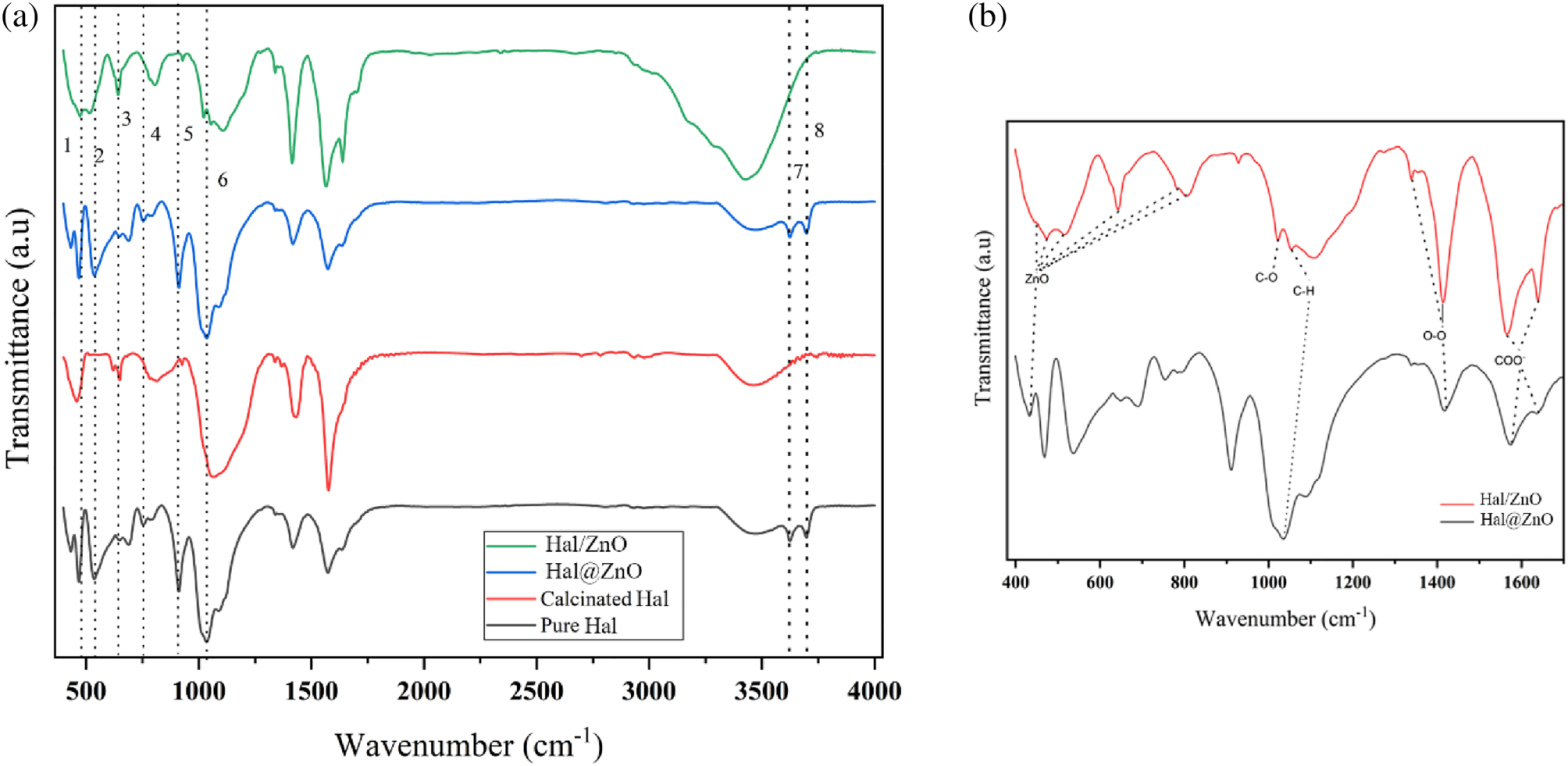
(**a**) FTIR spectra of pure Hal, calcined Hal, ZnO@Hal, and ZnO/Hal, (**b**) FTIR spectra of ZnO@Hal and ZnO/Hal at the 400–1800 cm^−1^ region.

The FTIR spectra of Hal, calcined Hal at 500 °C, ZnO@HAL, and ZnO/HAL are presented in Fig. 5a. As observed in Fig. 5a, a comparison between the pure HAL, ZnO@HAL, and ZnO/HAL showed new characteristic peaks in fingerprint region below 1000 cm^−1^ attributed to the ZnO stretching and vibrations, which confirm the formation of ZnO/HAL nanocomposite. Figure 5b depicts the FTIR spectra of ZnO@Hal and ZnO/Hal at the 400–1800 cm^−1^ region, corresponding to the symmetric and asymmetric stretching bonds of C–O, C–H, COO^−1^, and Zn–O bonding in the wurtzite structure of ZnO. The band at 1022 cm^−1^ is attributed to C–O stretching vibration. The characteristic peaks at 1563 and 1639 cm^−1^ are assigned to zinc carboxylate groups (COO^−1^)^41,42^. The band at 1055 cm^−1^ is characterized by C–H groups. The absorption bands at 440 cm^−1^, 516 cm^−1^, 640 cm^−1^, and 783 cm^−1^ are denoted by Zn–O stretching vibration. Additionally, the characteristic peaks at 3600–3700 cm^−1^ correspond to the deformation vibration of H_2_O molecules, which appear in the FTIR spectra of calcined HAL and ZnO/Hal. The FTIR spectrum of ZnO/Hal shows the broad absorption bands observed at 3200–3600 cm^−1^ attributed to the hydroxyl groups on the surface of ZnO. Similar results were reported by other researchers^43^. It should be noted that the FTIR spectrum of ZnO/Hal showed the peaks of the metaholloysite and that of ZnO.

### Zeta potential analysis

The Zeta potential of Hal, ZnO/Hal, and ZnO@Hal was studied to illustrate the surface charge change of Hal after ZnO coating on the halloysite, which affects the arsenic adsorption capacity of HAL. Table 2 shows the zeta potential results. As shown in Table 2, the zeta potential value of raw Hal was − 13.8 mV at a pH of 7 ± 0.2. The halloysite's point zero charges (pHpzc) were reported around 3^44^. Thus, it is expected that the zeta potential of raw Hal at neutral pH was negative. This phenomenon occurs due to the higher number of deprotonated silanol groups on the outer surface than the protonated aluminol groups on the inner surface at neutral pH. Additionally, according to Table 2, the zeta potential values of ZnO@Hal and ZnO/Hal have obtained − 32.7 mV and − 18.8 mV, respectively. These results showed that the deposition of ZnO on the halloysite increased the negative zeta potential of raw Hal due to the presence of hydroxyl groups on the surface of zinc oxide dispersed in an aqueous medium at neutral pH. In General, when ZnO is decorated on clay such as the halloysite, the amount of OH^−^ groups on the oxide surface will increase driving an enhancement of the negative charge of the clay surface^45^. It should be noted that the zeta potential below − 30 mV or beyond + 30 mV is considered as criteria for colloid stability (Haan et al. 2018). Therefore, the ZnO/Hal dispersion exhibits high stability because of the negative zeta potential compared to Hal in water^46,47^.

**Table 2:** The zeta potential of pure Hal and Hal nanocomposites.

Sample number	Zeta potential (mV)
Pure Hal	− 13.8 ± 1.5
ZnO@Hal	− 32.7 ± 1.6
ZnO/Hal	− 18.8 ± 0.7

### Thermogravimetric analysis (TGA)

**Figure 6:**
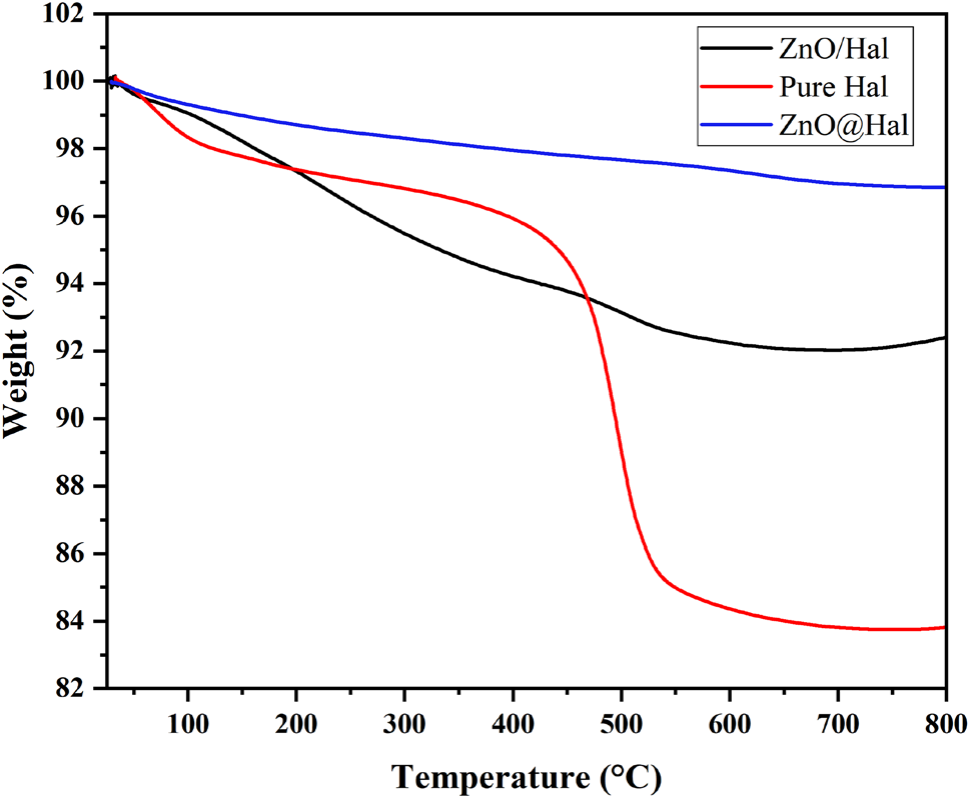
TGA graphs of pure Hal, ZnO@Hal, and ZnO/Hal.

The TGA graphs of pure Hal, ZnO@Hal, and ZnO/Hal are shown in Fig. 6. As can be seen, the major mass loss of HNT occurs at about 459 °C, which is attributed to its dehydroxylation of structural aluminum groups^45^. ZnO@Hal and ZnO/Hal were found to undergo only a very small weight loss when heated up to 800 °C. In pure Hal, ZnO@Hal, and ZnO/Hal, weight loss was approximately 17.7%, 7.6%, and 3.4%, respectively. As a result, the ZnO decoration of HNT could enhance its thermal stability.

### Morphology characterization

#### FESEM analysis

**Figure 7:**
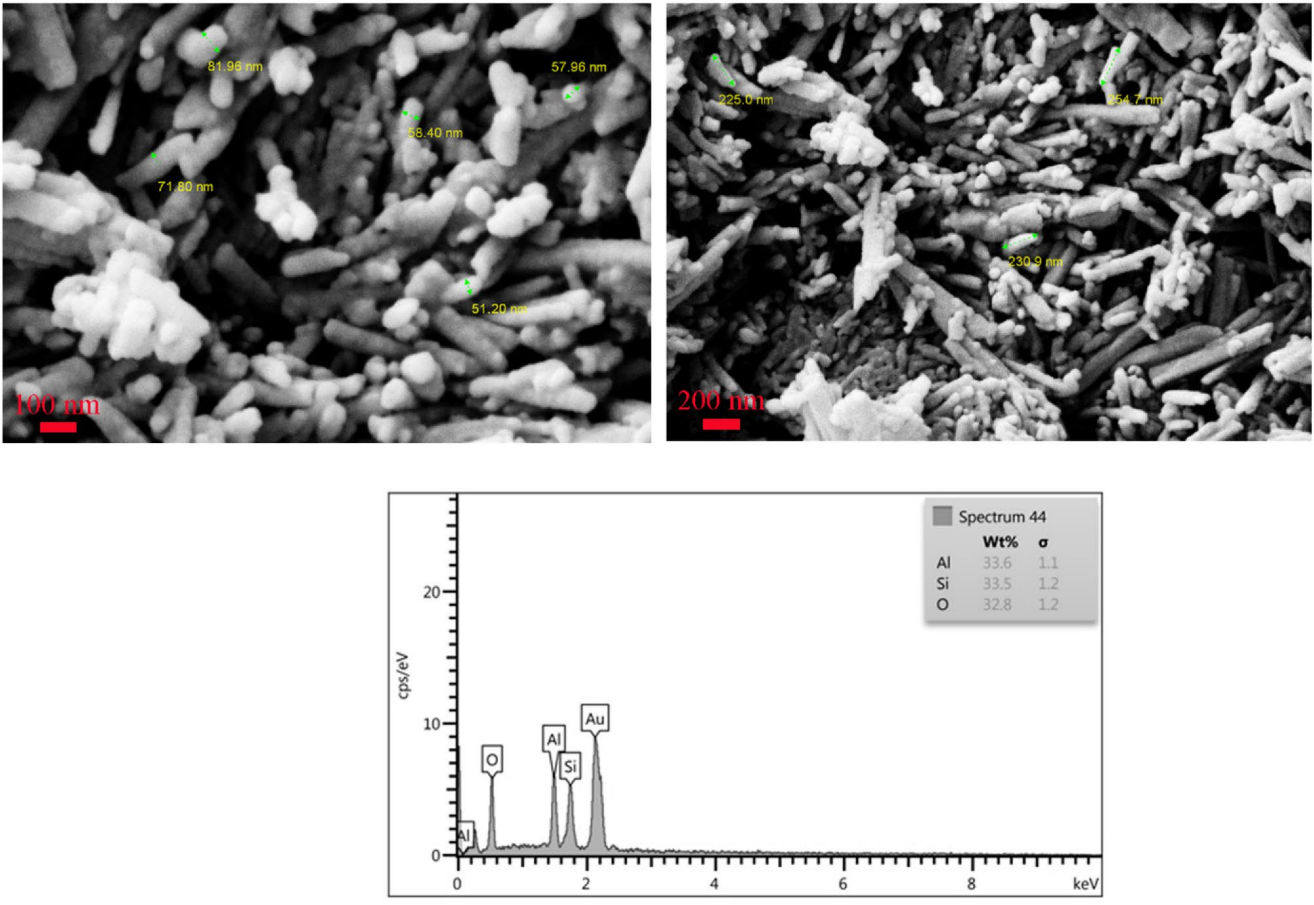
FESEM photos and the EDS spectrum of pure Hal.

**Figure 8:**
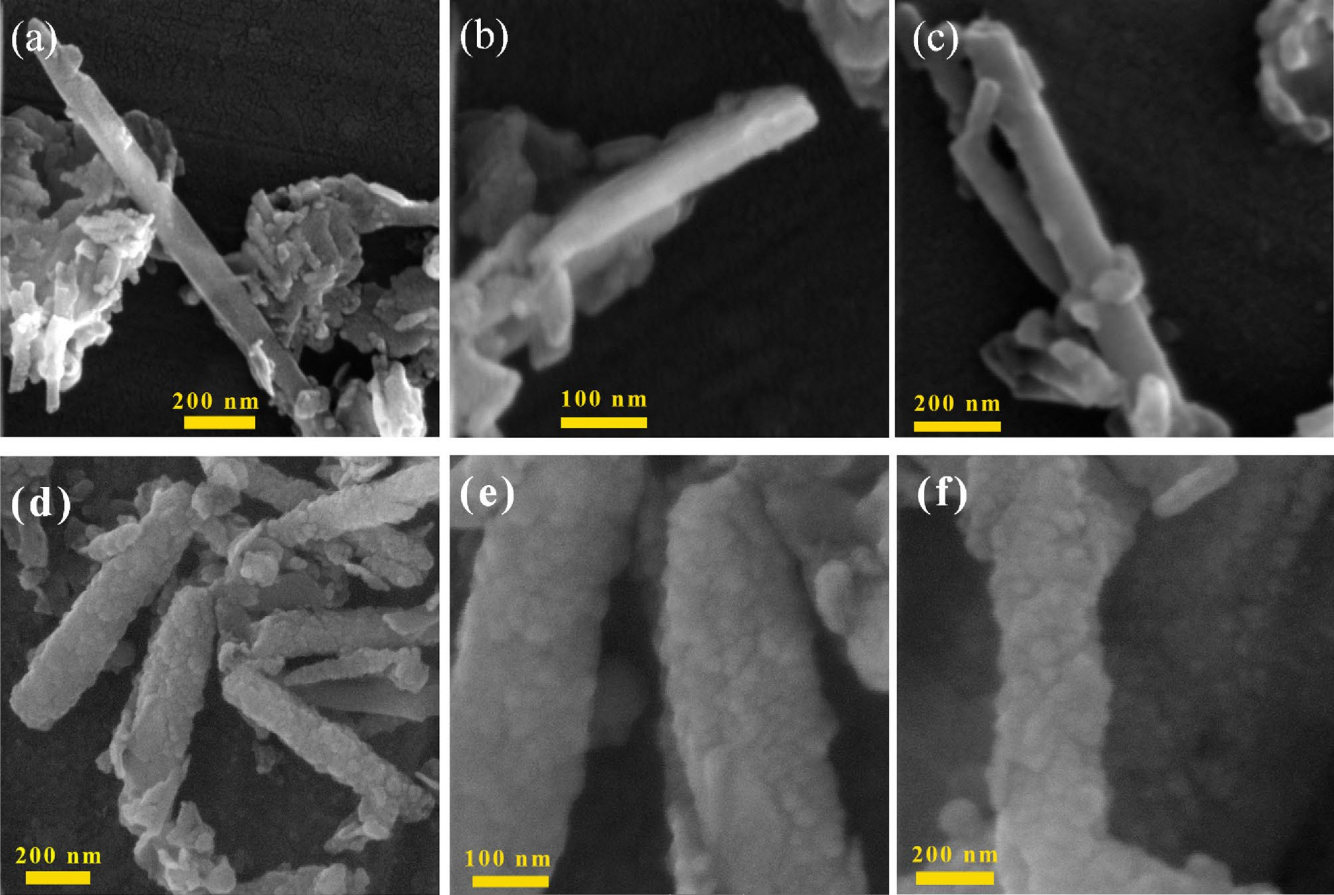
FESEM images of ZnO@Hal (**a**–**c**) and ZnO/Hal (**d**–**f**).

In order to gain a better understanding of morphology, FESEM analysis was performed at high magnifications. Figure 7 displays the FESEM images of Hal. As shown in Fig. 7a and b, the raw halloysite has a hollow cylindrical smooth structure with a length of up to 230 nm and an outer diameter of 55–100 nm. After the formation of the ZnO seed (ZnO@Hal) (Fig. 8a–c), the tubular morphology of the halloysite did not change significantly. However, its outer surface’s roughness has increased somewhat compared to the raw Hal. The presented EDS results in Table 3 confirmed the presence of Zn in the structure of ZnO@Hal. Figure 8d–f presents the FESEM images of ZnO/Hal. As shown in Fig. 8d–f, the ZnO nanoparticles are deposited on the ZnO@Hal, revealing a ZnO/Hal composite formation. The roughness of the surface and its complete coverage indicate the proper growth of zinc oxide by the HMTA agent with an initial molar ratio of 1:2 HMTA: Zn^2+^.The EDS results also confirmed an increase in the percentage of Zn in the ZnO/Hal composite compared to the ZnO@Hal. This result is in agreement with the zeta potential results (Table 2), which revealed that the surface of ZnO/Hal is more negative compared to that of ZnO@Hal.

**Table 3:** Energy dispersive X-ray spectroscopy (EDX) analysis of all samples.

Sample	Element	Wt%	Atomic %
ZnO@Hal	O	35.65	50.86
	Si	29.43	23.92
	Zn	8.63	3.03
	Al	26.24	22.19
ZnO/Hal	O	46.44	69.37
	Si	12.66	10.7
	Zn	31.45	11.49
	Al	9.51	8.42

#### TEM studies

**Figure 9:**
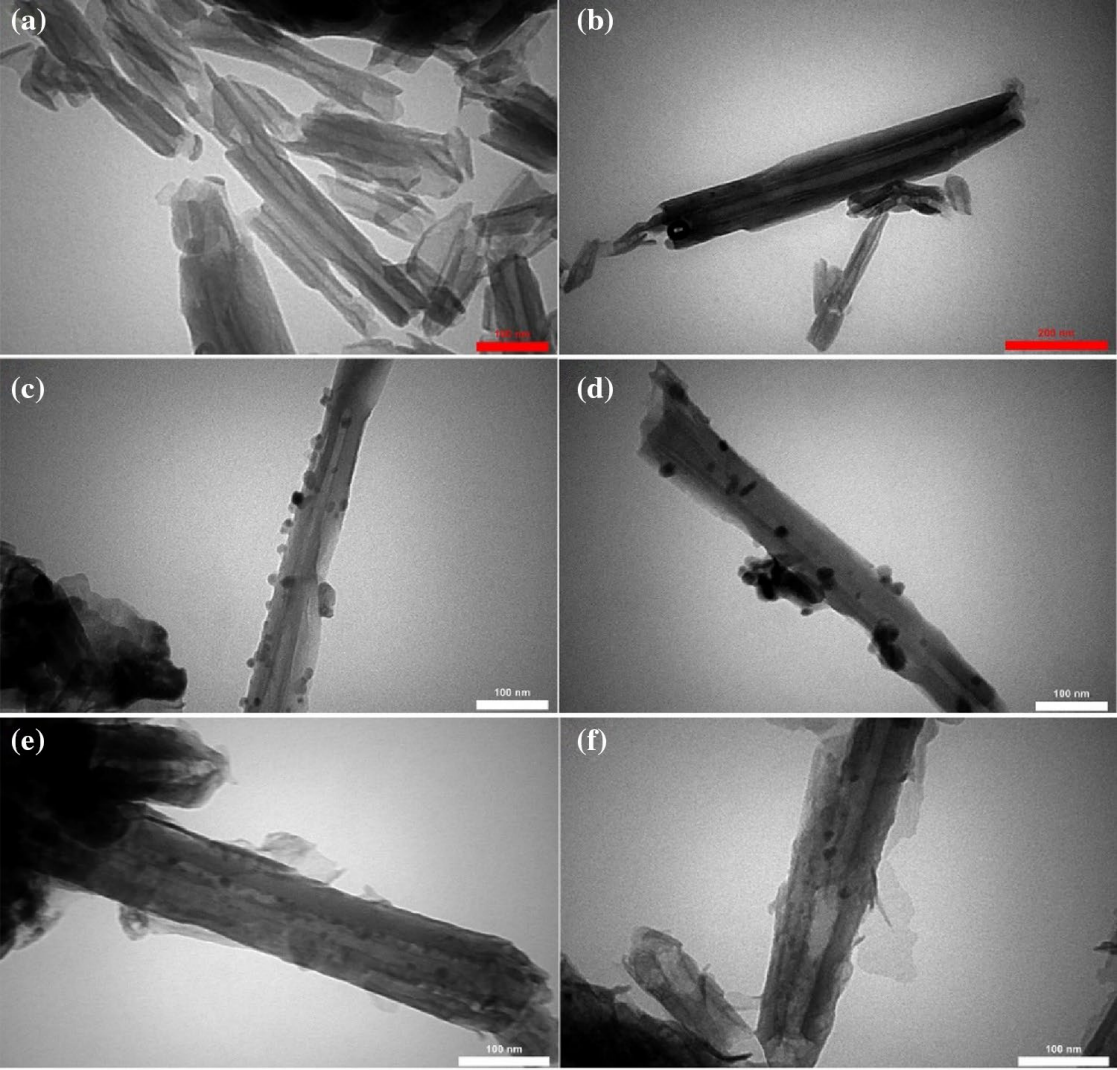
TEM graphs of raw Hal (**a**, **b**), seed growth of ZnO upon Hal surface (**c**, **d**), ZnO/Hal nanocomposites (**e**, **f**).

TEM analysis was used to further study the morphological features of raw Hal, ZnO@Hal, and ZnO/Hal. As shown in Fig. 9c and d, the ZnO nanoparticles were uniformly deposited on the outer surface of the halloysite and led to the formation of a rough dendritic structure. In contrast, the raw halloysite displays a smooth nanotubular structure (Fig. 9a,b). Additionally, the TEM image of ZnO@Hal (Fig. 9c,d) clearly shows that the ZnO nanoparticle is approximately hemispherical and binds to the outer surface of Hal. ZnO/Hal nanocomposite formation was also confirmed by TEM images (Fig. 9e,f). Generally, the Zn^+2^ ions are adsorbed on the halloysite's Si–O surface with a negative charge. Then, the nucleation and growth process is started at a calcination temperature of 500 °C^43,48^.

### Specific surface area studies (BET analysis)

**Figure 10:**
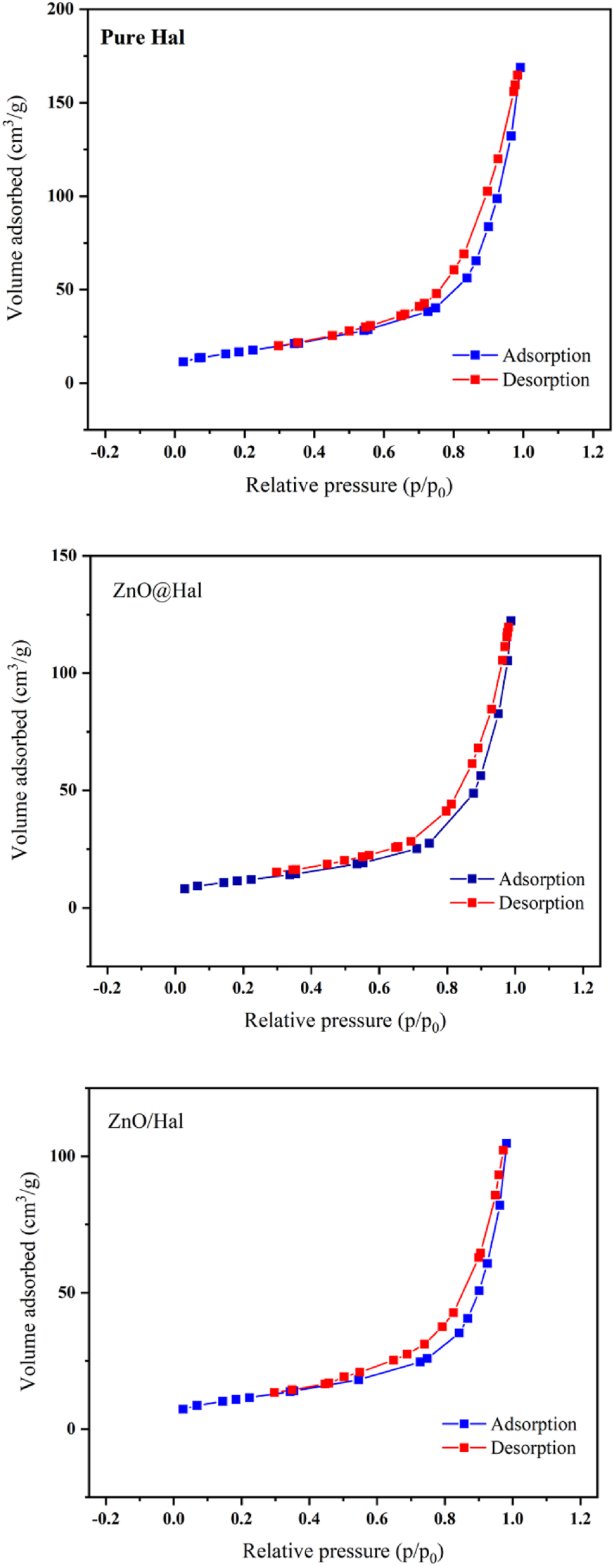
N_2_ adsorption–desorption isotherms of the pure Hal, ZnO@Hal, and ZnO/Hal.

The N_2_ adsorption–desorption analysis (the Barratt–Junior–Hellenda (BET) and Barratt–Junior–Hellenda (BJH) analyses) was performed to identify the surface structure, including specific surface area, pore size, pore volume, pore distribution and porosity features of raw Hal, ZnO@Hal, and ZnO/Hal nanocomposites. Figure 10 illustrates the N_2_ adsorption/desorption isotherms of pristine Hal and prepared samples. As shown in Fig. 10, all samples presented a type IV according to the IUPAC classification. The results are summarized in Table 4. It can be seen that Hal exhibited the largest surface area (61.2 m^2^/g) compared to ZnO@Hal (41.3 m^2^/g) and ZnO/Hal nanocomposite (39.9 m^2^/g). In addition, the pore volume of Hal (14 cm^3^/g) is decreased after seed growth of ZnO (ZnO@Hal = 9.4 cm^3^/g) and formation of ZnO/Hal nanocomposite (ZnO/Hal = 9.18 cm^3^/g). Other researchers reported similar results^49^. According to the literature, the specific surface area of pure ZnO is approximately 13 m^2^/g, and the surface area and pore volume of ZnO/halloysite nanocomposites are considerably smaller than the pure halloysite. Covering the lumen of Halloysite with ZnO nanoparticles may decrease the number of macropores and consequently reduce the pore volume and specific surface area of ZnO/Hal nanocomposite. Overall, Halloysite with a high specific surface area has excellent potential to support ZnO nanoparticles^50–52^.

**Table 4:** Specific surface area (SS_A_), pore-volume (V_p_), and pore-size (D_av_) data.

Sample	SS_A_ (m^2^/g)	V_p_ (cm^3^/g)	D_av_ (nm)
Hal	61.2	14	16.8
ZnO@Hal	41.3	9.4	18.3
ZnO/Hal	39.9	9.2	16.2

### The adsorption performance of halloysite nanocomposites for arsenite (As(III)) removal

The adsorption capacity of Hal, and prepared Hal nanocomposites was evaluated by removing 50 ppm of arsenite. Table 5 presents the adsorption conditions and obtained results. It should be noted that all adsorption experiments were performed on the neutral pH because of the amphoteric nature of ZnO. The ZnO nanoparticles dissolved under acidic and alkaline environments. However, the dissolution rate of ZnO is faster under acidic media than in weakly alkaline environments. As shown in Table 5, the removal efficiency of As(III) is increased from 5% for pure Hal to 23% in 24 h (as the maximum time) for ZnO@Hal. In addition, the highest removal efficiency is related to ZnO/Hal nanocomposite, equal to 76% in 24 h. The adsorption mechanism of As(III) by adsorbents involves physical and chemical interactions between the adsorbate and the adsorbent surface. The mechanism of As(III) removal can be in effect attraction of As(III) ions with the boundary layer of the adsorbent through electrostatic attraction forces and the formation of complexes in the presence of one water molecule between As(III) and adsorbent functional groups like hydroxyl group^41,49,53^. According to the BET and zeta potential results, it can be concluded that the increased As(III) adsorption is mainly due to the increased negative potential on the tube surface of Hal and Hal nanocomposites. In other words, the adsorption of cationic As(III) more probably occurs on the external surface of halloysite nanotubes.

**Table 5:** As(III) adsorption conditions and obtained results.

Sample	C_0_ (mg/L)	C_e_ (mg/L)	V (lit)	m (g)	Removal efficiency (%)
Pure Hal	50.2 ± 0.1	47.6 ± 0.1	0.04	0.1	5
ZnO NPs	50.2 ± 0.1	33.9 ± 0.1	0.04	0.1	37
ZnO@Hal	50.2 ± 0.1	38.6 ± 0.1	0.04	0.1	23
ZnO/Hal	50.4 ± 0.2	29.7 ± 0.1	0.04	0.1	76

#### Effect of contact time on the removal efficiency

**Figure 11:**
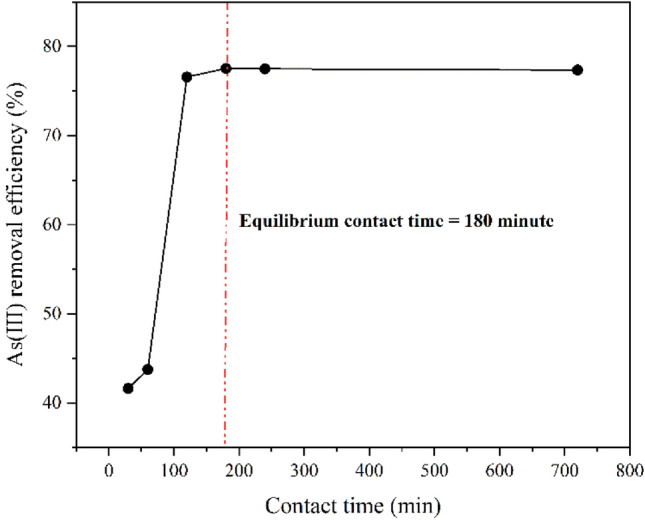
Effect of contact time on the removal efficiency of As(III). (T = 298°K, Adsorbent dosage = 2.5 g/L and C_0_ = 50 mg/L).

The effect of contact time on the removal efficiency and adsorption capacity of ZnO/Hal at an initial As (III) concentration of 50 mg/L was investigated. The parameters of adsorbent dosage (2.5 g/L) and temperature (298 °C) were kept constant. As shown in Fig. 11, in the early stages, the removal efficiency of As(III) increases significantly due to various active sites, which increases with an increase in contact time from 120 to 180 min and then changes slightly. According to the results, the equilibrium contact time was 3 h.

#### Effect of initial pH on As(III) removal

**Figure 12:**
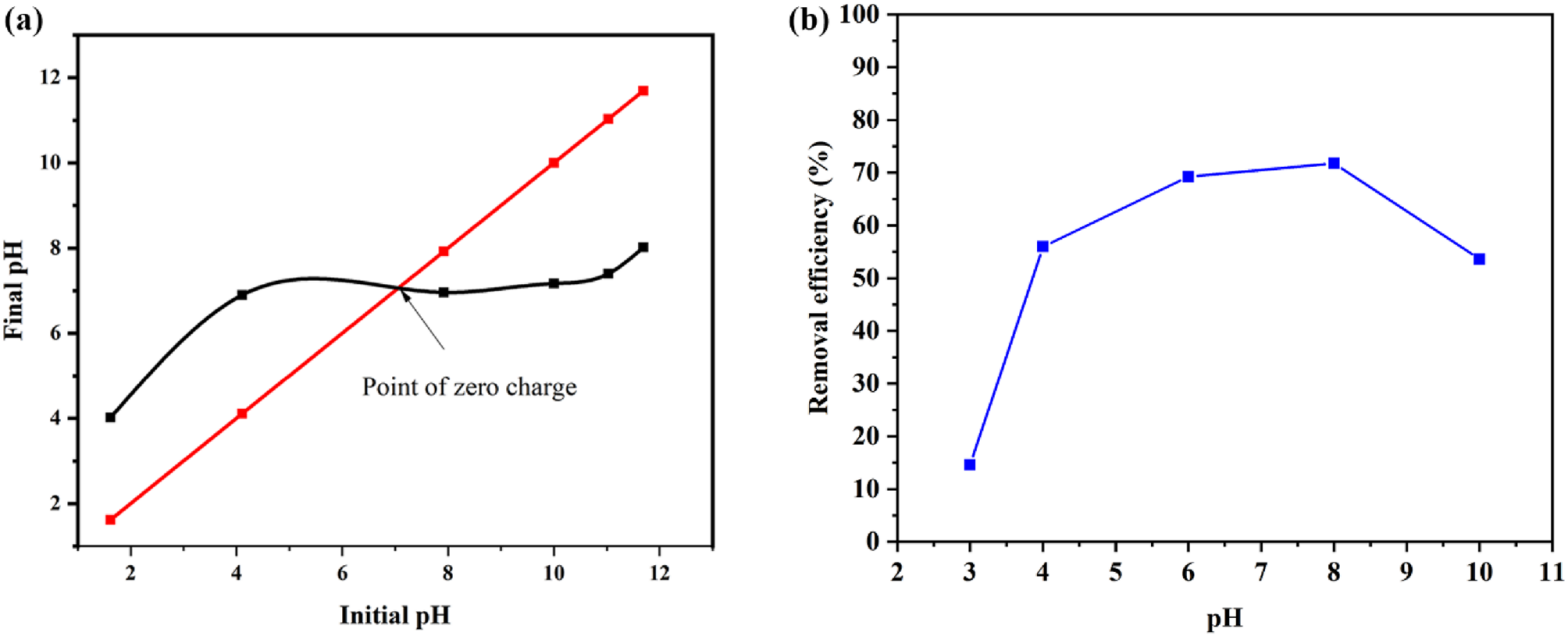
The PZC for ZnO/Hal sample (**a**), the effect of solution pH on the removal efficiency of As(III) onto ZnO/Hal sample (experimental conditions: adsorbent dose: 2.5 g/L As(III) initial concentration: 50 mg/L, temperature: 298 K, contact time: 240 min).

The pH of the solution determines the chemical speciation of metal ions, the degree of ionization on the adsorbent surface, the charge, and the surface chemistry of the adsorbent surface. Considering ZnO's instability in strongly acidic and alkaline solutions, the effect of pH on As(III) removal efficiency has been studied between pH 3 and pH 10. NaOH (1 M) or HCl (1 M) solutions were added to adjust As (III) solution pH. According to the results (Fig. 12b), As(III) removal efficiency increased from pH 3 to 8 and then decreased to pH 10. The most common As(III) state is the non-ionic state, (H_3_ AsO_3_), at pH values less than 9.2^54^. In addition, H_2_AsO_3_^−^ is also present in the solution at near pH 9^55^. Solid-addition technique was used to measure pHpzc^56^. The results are presented in Fig. 12a. Based on Fig. 12a, it can be concluded that ZnO/Hal has a pHpzc value of nearly 6.9, meaning that the surface is negatively charged when the pH is higher than observed and vice versa^57^. Because of this, adsorbent surfaces become protonated at pH levels less than 6.9, which leads to a greater removal of As(III) via electrostatic attraction between the positively charged surface and the negatively charged H_2_AsO_3_^−^ molecules^58^. In addition, adsorption occurs between non-ionic As (H_3_AsO_3_) and surface functional groups of ZnO/Hal in the pH range of 4–8 through the formation of surface complexes^55^. Between pH 8 and 10, electrostatic repulsion between negatively charged ZnO/Hal surfaces and deprotonated anionic arsenic led to a sharp decrease in removal efficiency. Previous studies also reported similar results^59^.

#### Effect of initial concentration

**Figure 13:**
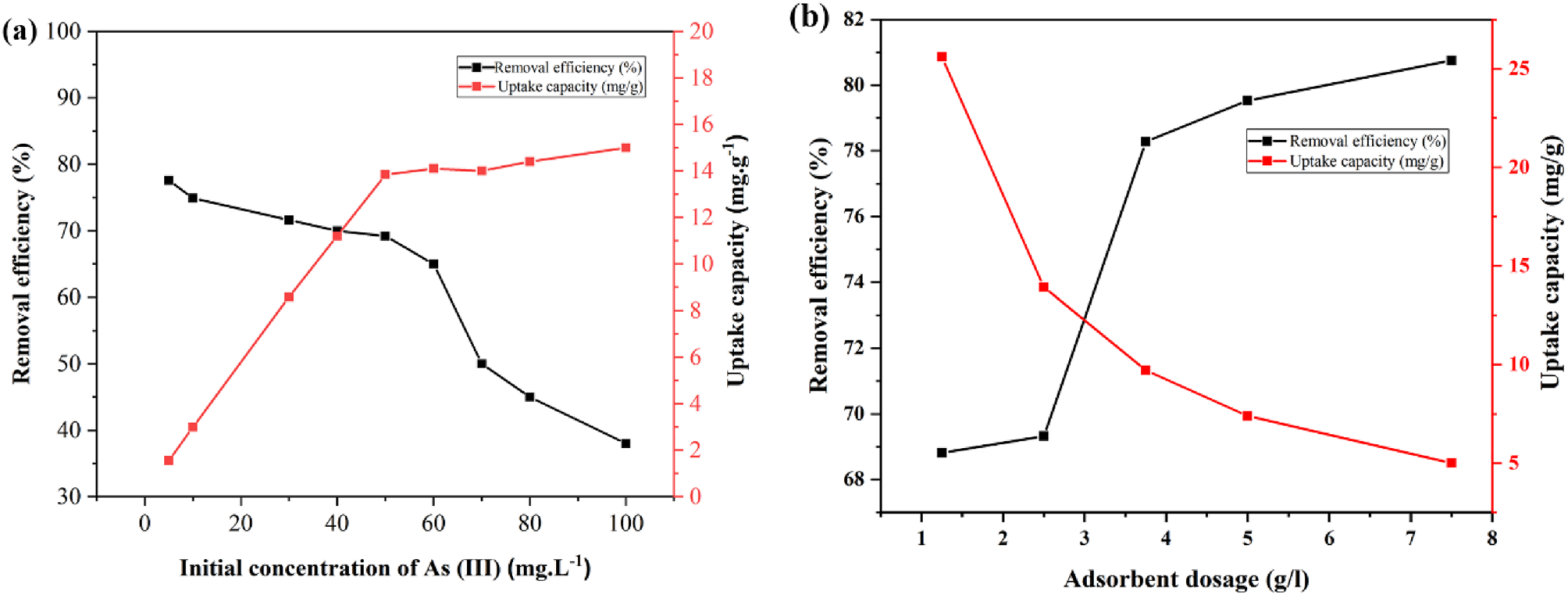
(**a**) The effect of initial concentration of As (III) on removal efficiency and adsorption capacity (q_e_) (T: 298°K, adsorbent dosage: 2.5 g/L, contact time: 3 h) and (**b**) Effect of adsorbent dosage on the removal of As (III) (T = 298°K, t = 180 min and C_0_ = 50 mg/L).

The effect of the initial concentration of As(III) on the removal efficiency and adsorption capacity of As(III) onto the sample ZnO/Hal was investigated by changing As(III) concentration from 5 to 100 mg/L and the obtained results are displayed in Fig. 13a. The results showed that the removal efficiency decreased significantly with an increase in the initial concentration of As(III) from 5 to 100 mg/L. In contrast, the adsorption capacity increased with an increase in the initial concentration of As(III) from 5 to 50 mg/L and became saturated at 50 mg/L of As(III), and a further increase in the initial concentration of As(III) does not remarkably change the adsorption capacity. The observed phenomenon can be explained by an excessive number of As(III) molecules compared to the number of available adsorption sites at higher concentrations of As(III) or by an increase in cohesion forces between As(III) molecules.

#### Effect of adsorbent dosage

Effect of adsorbent dosage on As(III) removal onto the sample ZnO/Hal was evaluated. The obtained results are shown in Fig. 13b. The results confirmed the removal efficiency is enhanced with an increment in adsorbent dosage, while the adsorption capacity is decreased. Due to the increased surface area and availability of active sites, increasing the adsorbent dosage generally improves removal efficiency. Moreover, increases in adsorption dosage, due to unsaturation of some adsorption sites and decreasing active sites due to aggregation processes, reduce the adsorption capacity^34^.

### Effect of temperature and thermodynamic parameters

**Figure 14:**
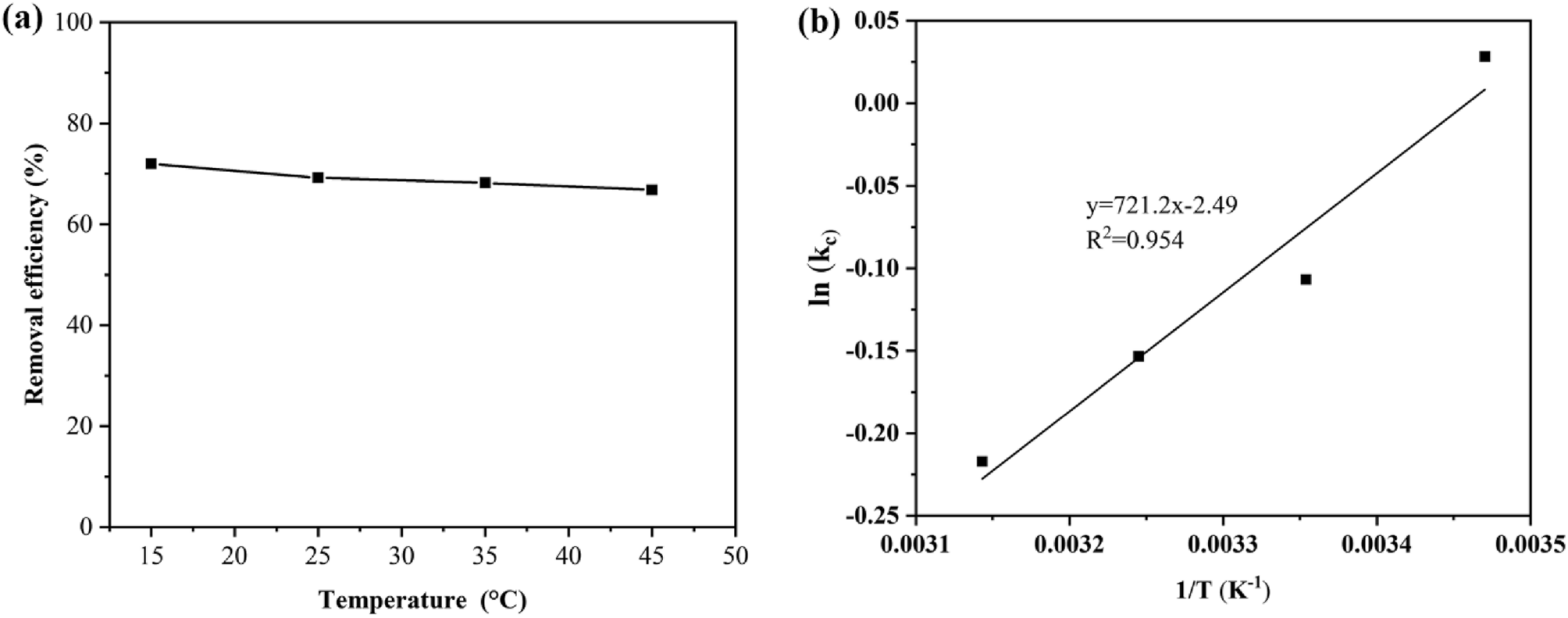
Effect of temperature on As(III) adsorption (**a**), the linear plot of Thermodynamic (**b**).

Temperature effects on As(III) adsorption were investigated at 15, 25, 35, and 45 °C. Adsorption was carried out in a refrigerated incubator shaker apparatus. As shown in Fig. 14a, temperature increases of 15 °C to 25 °C resulted in a 3% decrease in removal efficiency, and further increases had no significant impact. Adsorption feasibility was evaluated using thermodynamic studies^60^. To evaluate the thermodynamics of the As(III) adsorption process, the changes in enthalpy (ΔH°), entropy (ΔS°), and free energy (ΔG°) were determined using the following equations^61^:

3 $${\mathrm{K}}_{\mathrm{c}}=\frac{{q}_{e}}{{\mathrm{c}}_{e}}$$

4 $$\mathrm{\Delta G}^\circ =-{\mathrm{RTlnK}}_{\mathrm{c}}$$

5 $$\mathrm{ln}\left({\mathrm{K}}_{\mathrm{C}}\right)=-\left(\frac{{\Delta \mathrm{H}}^{0}}{\mathrm{RT}}\right)+\left(\frac{{\Delta \mathrm{S}}^{0}}{\mathrm{R}}\right)$$

R (8.314 J mol^−1^ K^−1^) represents the universal gas constant, k_c_ is the distribution coefficient, and T (K) is the temperature. The linear plot of thermodynamics is shown in Fig. 14b. The values of ΔH, ΔS, and ΔG at different temperatures were calculated from slope and intercept by the linear plot and presented in Table 6. The obtained negative ΔG° value at temperature 15 °C confirmed the spontaneity and feasibility of the As(III) adsorption process at this temperature^62,63^. However, the positive ΔG° values at 25, 35, and 45 °C indicate that spontaneity isn't favorably favored at these temperatures. In addition, As(III) adsorption on ZnO/Hal was exothermic, as indicated by the negative value of ΔH^64^. The negative entropy (ΔS°) value indicates that there are minor variations in the internal structure of the adsorbent and that there are fewer random interactions at the interface between the solid and the solution^65^.

**Table 6:** Thermodynamic parameters of the adsorption of As(III) on ZnO/Hal at various temperatures.

Adsorbent	T(K)	K_C_	Δ*G*° (kJ mol^−1^)	Δ*H*° (kJ mol^−1^)	Δ*S*° (kJ mol^−1^ K^−1^)	R^2^
ZnO/Hal	288.15	1.028571	− 0.06749	− 23.73	− 0.0207	0.95
	298.15	0.898701	0.264749			
	308.15	0.857862	0.39278			
	318.15	0.804819	0.57435			

#### Adsorption isotherm

**Figure 15:**
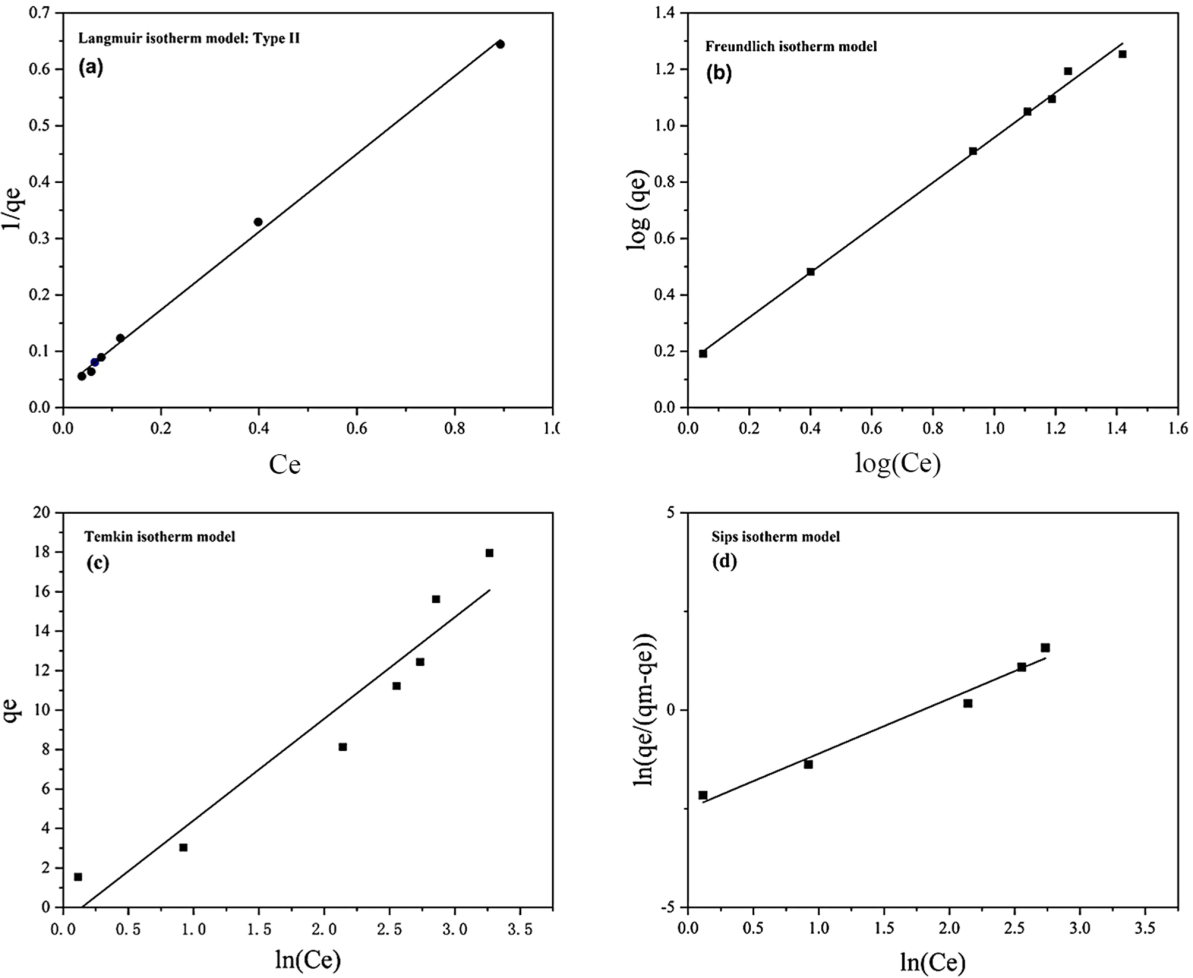
The As(III) adsorption linear isotherms on ZnO/Hal; (**a**) Langmuir isotherm, (**b**) Freundlich isotherm, (**c**) Temkin isotherm, and (**d**) Sips isotherm.

**Figure 16:**
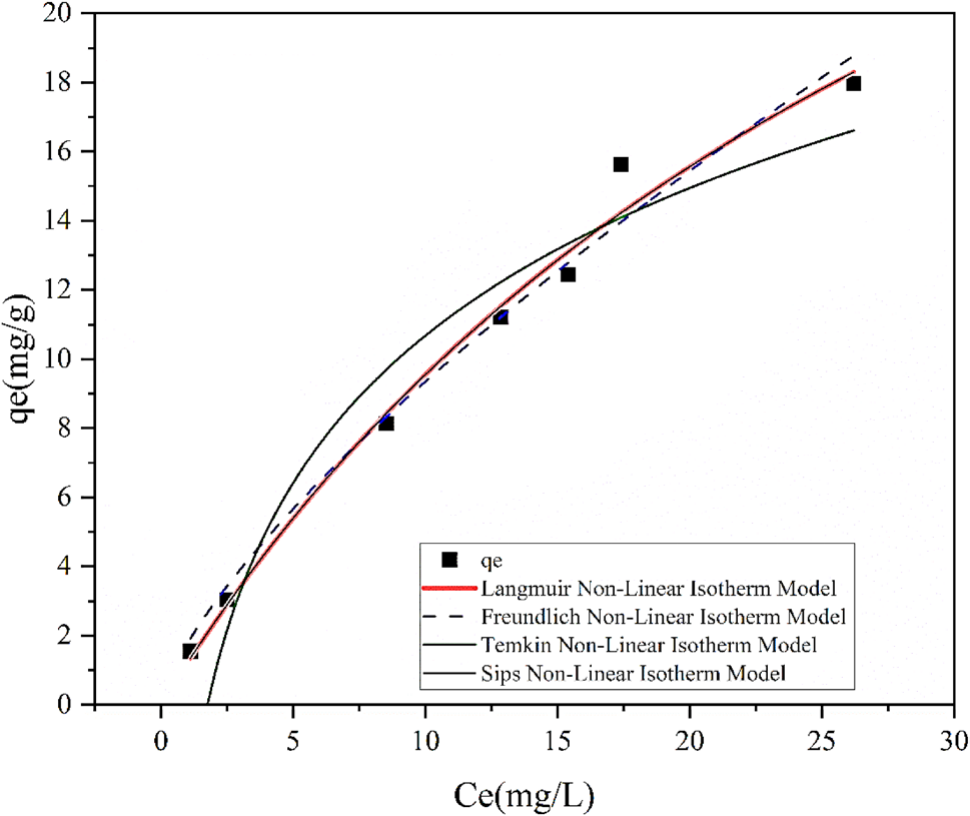
The As(III) adsorption nonlinear isotherms on ZnO/Hal; Langmuir, Freundlich, Temkin Isotherm and Sips Isotherms.

The adsorption isotherms provide information about the mechanism of sorption as well as the interaction between adsorbates molecules and adsorbents at constant temperatures. In this study, different isotherm models such as Langmuir (type II), Freundlich, Temkin, and Sips in linear and non-linear forms were considered to fit the equilibrium data of As (III) onto ZnO/Hal nanocomposite. The linear and non-linear equations and related parameters are given in Table 7. According to the obtained correlation coefficients for linear equations (Fig. 15 and Table 7), the experimental data are better fitted with Langmuir (type II) and Freundlich isotherms compared to Temkin and Sips models^66–68^. Equilibrium adsorption data are usually fitted to linear isotherm models. Non-linear models (Fig. 16) could describe equilibrium data better than linear ones. Non-linear models are generally transformed into linear models by altering their error structures, which may violate standard least squares' error variance and normality assumptions. Therefore, non-linear equations were also investigated in this study. Based on the parameter estimates for non-linear isotherm models (Table 7), the Langmuir model fits equilibrium data better than the Freundlich and Temkin models. The maximum As(III) uptake capacity was achieved at 42.07 mg/g according to the non-linear Langmuir (type II) model. Moreover, the Sips model, which combines Langmuir and Freundlich models, is the best-fit model alongside Longmuir with a high correlation coefficient (R^2^ = 0.98). Langmuir's model is suitable for monolayer adsorption since each vacant site is occupied by a single molecule that has been adsorbed. The Freundlich is applied to multilayer adsorption on the adsorbent's surface^34,69,70^.

**Table 7:** Non-linear and linearized equations for Langmuir, Freundlich, Temkin, and Sips isotherm models and related constants and correlation coefficients for As(III) removal efficiency on the ZnO/Hal.

Isotherm model	Non-linear equation	Linear equation	Parameter	Linear equation	Non-linear equation
Langmuir isotherm (type II)	${q}_{e}=\frac{{q}_{m}{K}_{l}Ce}{1+{K}_{l}{C}_{e}}$	$\frac{1}{{q}_{e}}=\left(\frac{1}{{K}_{l}{q}_{m}}\right)\frac{1}{{C}_{e}}+\frac{1}{{q}_{m}}$	q_m_: maximum adsorption capacity	2.83	42.07
			k_l_: Langmuir isotherm constant	0.51	0.029
			R^2^: correlation coefficient	0.99	0.98
Freundlich isotherm	${q}_{e}={K}_{f}{C}_{e}^\frac{1}{n}$	$\mathrm{log}\left({q}_{e}\right)=\mathrm{log}\left({K}_{f}\right)+\frac{1}{n}\mathrm{log}({C}_{e})$	K_f_: Freundlich isotherm constant	1.44	1.76
			1/n: Adsorption intensity	0.79	0.72
			R^2^: correlation coefficient	0.99	0.97
Temkin isotherm	${q}_{e}=\frac{RT}{b}\mathrm{ln}\left({{K}_{T}C}_{e}\right)$	${q}_{e}=\frac{RT}{b}\mathrm{ln}\left({C}_{e}\right)+\frac{RT}{b}\mathrm{ln}\left({K}_{T}\right)$	K_T_: Temkin isotherm equilibrium constant	0.86	0.56
			b: Temkin isotherm constant	481.3	402
			R^2^: adsorption intensity	0.92	0.93
Sips isotherm	${q}_{e}=\frac{{q}_{m}{{(K}_{Si}Ce)}^{n}}{1+{{(K}_{Si}Ce)}^{n}}$	$\mathrm{ln}(\frac{{q}_{e}}{{q}_{m}-{q}_{e}})=\frac{1}{n}\mathrm{ln}\left({C}_{e}\right)+\mathrm{ln}\left({K}_{Si}\right)$	q_m_: maximum adsorption capacity	15.79	42.5
			K_si_: Sips isotherm constant	0.167	0.028
			R^2^: correlation coefficient	0.97	0.98
			1/n: Sips exponent	1.39	0.99

#### Adsorption kinetic and mechanism

**Figure 17:**
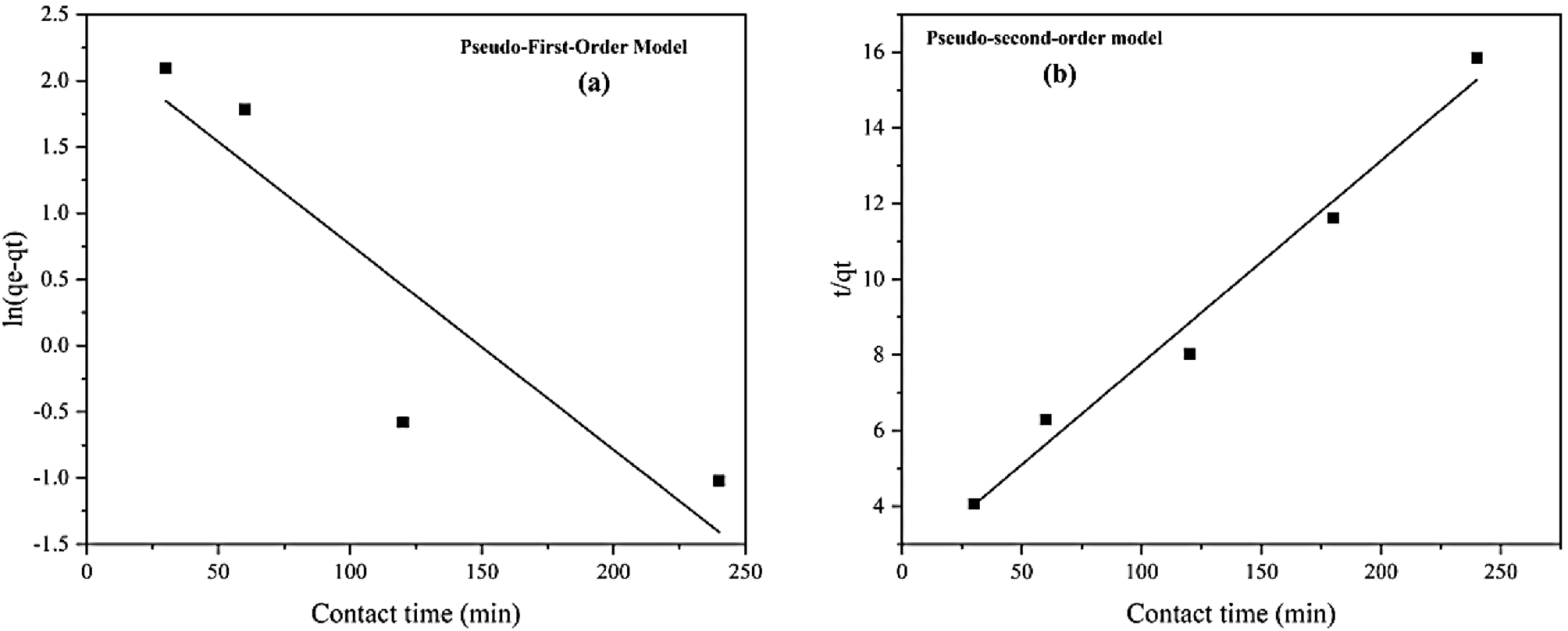
The As(III) adsorption linear kinetic model on ZnO/Hal; (**a**) Pseudo-first-order model, (**b**) Pseudo-Second-order model.

Adsorption kinetics models predict reaction times and explain the adsorption mechanism. In this study, the adsorption kinetics of As(III) upon ZnO/Hal nanocomposite were investigated by linear pseudo-first-order (PFO) and pseudo-second-order (PSO) models, which are illustrated in Table 8. It is generally assumed that adsorption is a physical process in the pseudo-first-order model controlled by mass transfer from the adsorbate to the surface. In comparison, the pseudo-second-order model explains chemisorption as the dominant adsorption process^71^. Figure 17 shows the plots of pseudo-first-order and pseudo-second-order adsorption kinetic models. According to the results, experimental data agree with the PSO model with a higher R^2^ value than the PFO model.

**Table 8:** Parameters of pseudo-first-order and pseudo-second-order constants obtained for the adsorption of As(III) using ZnO/Hal nanocomposite.

Isotherm model	Linear equation	Parameter	Description	Result
Pseudo-first-order model (PFO)	$\mathrm{ln}\left({q}_{e}-{q}_{t}\right)=\mathrm{ln}{q}_{e}-{k}_{1}t$	q_e_	Equilibrium adsorption capacity	10.10
		k_1_	Rate constant (min^−1^)	− 0.0155
		R^2^	Egression coefficients	0.81
Pseudo-second-order model (PSO)	$\frac{t}{{q}_{t}}=\frac{1}{{k}_{2}{q}_{e}^{2}}+\frac{1}{{q}_{e}}t$	q_e_	Equilibrium adsorption capacity	18.67
		k_2_	Rate constant (min^−1^)	0.0011
		R^2^	Egression coefficients	0.98

According to the PSO equation, the equilibrium adsorption capacity (qe) is 18.67 mg/g, close to the experimental value (15.49 mg/g). In contrast, the computed qe value for the PFO model (10.10 mg/g) is greatly differs from the observed q_e_ value. Therefore, the chemisorption is a controlling mechanism for the adsorption of As(III) upon ZnO/Hal. A chemisorption process involves valency forces when electrons are shared or exchanged between an adsorbent and adsorbate^33,72,73^. Other researchers have reported similar results in As (III) adsorption on various adsorbents^30,32,34,69,70,73,74^.

**Figure 18:**
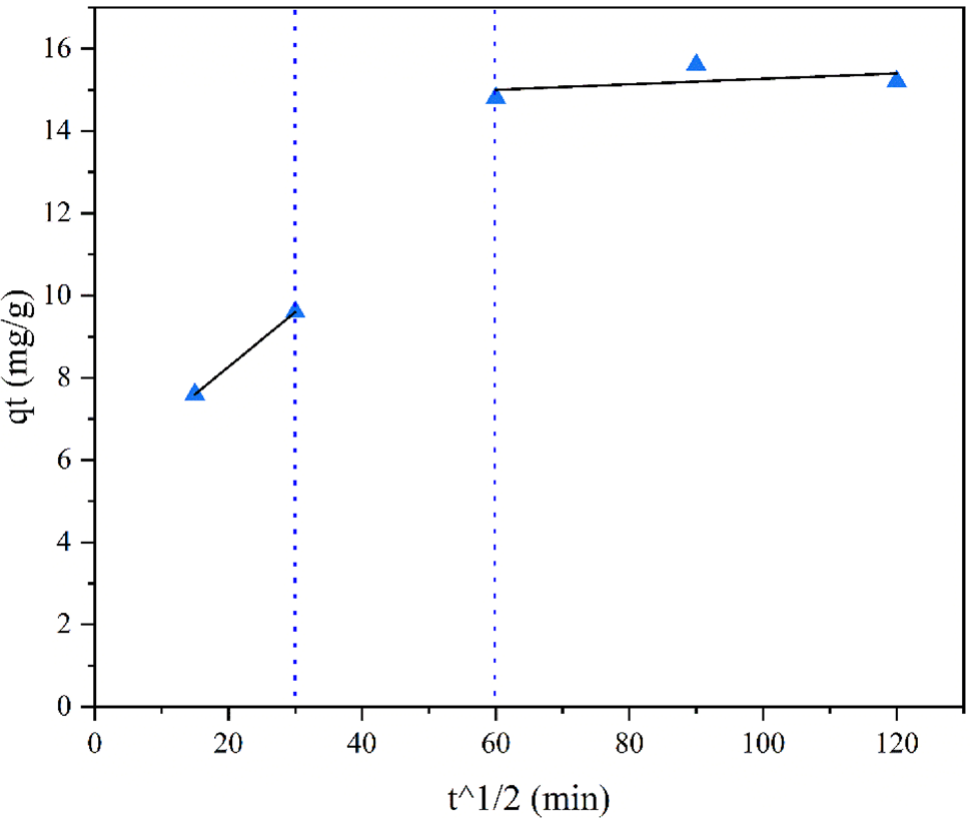
Weber and Morris intraparticle diffusion model with two sections for As(III) adsorption on the ZnO/Hal nanocomposite.

Intra-particle diffusion model (IPD) Eq. (6) was also used to study the mechanism of As(III) adsorption onto ZnO/Hal nanocomposite^75^.

6 $${q}_{t}={K}_{IPD}{t}^{1/2}+C$$

where, C is an arbitrary constant represents the boundary layer thickness. If C is zero, the linear line should pass through the origin. Consequently, film diffusion could be ignored because of a lack of thickness or a shallow thickness. Therefore, the rate-controlling step remains intraparticle diffusion throughout the entire adsorption kinetics. Table 9 and Fig. 18 present the results. The results showed no zero intercepts for q_t_ versus t^1/2^ (Table 9). As a result, both intraparticle diffusion and film diffusion are involved in the rate-limiting step. There can be a difference in the mass transfer rate between the adsorption stages, which can cause straight lines to deviate from the origin. A higher K_IPD_ value for As (III) at initial concentration indicates appropriate film diffusion from the arsenic bulk solution to ZnO/Hal nanocomposite due to increased electrostatic forces between the adsorbate and the adsorbent surface. As a result, the higher C for As(III) demonstrated that As(III) removal was primarily associated with surface adsorption.

**Table 9:** Diffusion parameters for the Weber and Morris intraparticle model for As(III) in single and mixed solutions.

Initial concentration (mg/L)	Section 1			Section 2		
	K_IPD_	R^2^	C	K_IPD_	R^2^	C
50	0.066	1	5.6	0.0033	0.5	14.6

#### Regeneration and reuse of ZnO/Hal adsorbent

**Figure 19:**
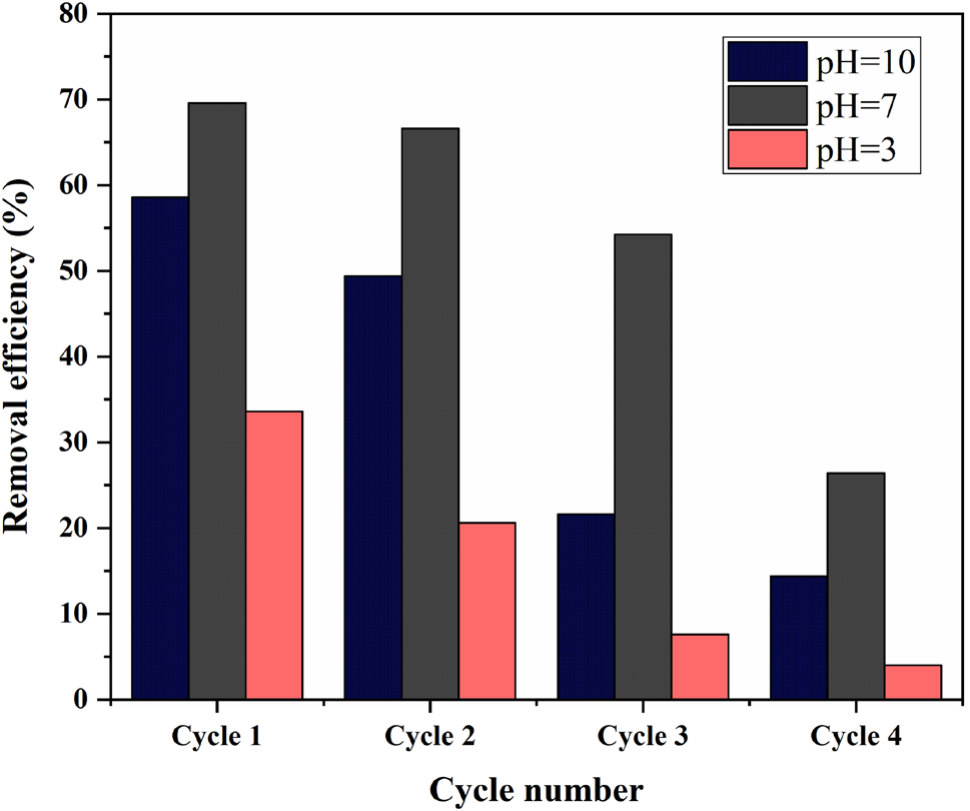
Reusability of ZnO/Hal adsorbent with aqueous solutions with different pH (experimental conditions: T: 298°K, adsorbent dosage: 2.5 g/L, contact time: 3 h, initial concentration of As(III): 50 ppm).

Regenerating exhausted adsorbents is crucial to maximizing efficiency and minimizing operating costs^76^. However, the stability of the adsorbent is crucial in determining the effectiveness of the regeneration process^63^. The chemical regeneration process is the most efficient method for desorbing a particular species from surface adsorbents in solutions. Adsorbent regeneration efficiency depends on solution pH and can be enhanced by changing pH of the solution^77,78^. Hydrochloric acid and sodium hydroxide are commonly used to change the pH of solutions. In this study, HCl (1 M) and NaOH (1M) were used to adjust the solution pH and study the desorption of As(III) from ZnO/Hal sample. For this purpose, 2.5 g/L of the used adsorbent was in contact with an aqueous solution with a different pH for 1 h under constant stirring. Next, the samples were repeatedly rinsed with deionized water and dried at 100 °C for 4 h. The recovered materials were then exposed to the same experimental conditions for adsorption experiments. As(III) removal efficiency against adsorption–desorption cycles is shown in Fig. 19. Increased regeneration cycles reduced As(III) removal efficiency, particularly when aqueous solutions with pH 10 were used to regenerate the adsorbent. Moreover, the As(III) removal efficiency of recovered adsorbents in pH 3 aqueous solution declined from 59 to 14% after five adsorption–desorption cycles. The reduction was 76% to 26% for recovered adsorbents in pH 7 aqueous solution, and 34% to 4% for regenerated ones in pH 10. There is a possibility that the adsorption–desorption cycle degrades the ZnO structure, destroying the active adsorption sites, and resulting in a decrease in adsorption. Based on the results of five cycles of adsorption and desorption, ZnO/Hal regenerated using neutral aqueous solutions showed high adsorption efficiency.

## Conclusion

Halloysite clay with a length of approximately 230 nm and an outer diameter in the range of 55–100 nm has been applied as a porous substrate for the loading of ZnO. The prepared ZnO/Hal nanocomposite with HMTA: Zn^+2^ initial molar ratio of 1:2 indicated appropriate ZnO nucleation and growth upon halloysite. The Hal nanocomposite exhibited the best As(III) adsorption performance with a removal efficiency of 76%, fifteen times the pure Hal. The surface structure and its surface chemistry analyses showed that although the specific surface area of the prepared Hal nanocomposite was lower than the pure Hal, the absorption efficiency increased due to the more negative zeta potential of the nanocomposite sample. The equilibrium contact time of 180 min was obtained for ZnO/Hal nanocomposite. It was found that the non-linear Langmuir (type II) and Sips isotherm models provided a perfect fit to the experimental data, indicating maximum adsorption capacities of 42.07 mg/g and 42.5 mg/g, respectively. According to the kinetic studies, experimental data were in agreement with the PSO model.

## Data Availability

The data of this study will be available upon reasonable request via contacting the corresponding author (norouzbeigi@iust.ac.ir).
